# Focus on Cardiologic Findings in 30 Children With PANS/PANDAS: An Italian Single-Center Observational Study

**DOI:** 10.3389/fped.2019.00395

**Published:** 2019-10-01

**Authors:** Manuel Murciano, Davide Maria Biancone, Giulia Capata, Isabella Tristano, Vanessa Martucci, Cristiana Alessia Guido, Silvia Anaclerio, Lorenzo Loffredo, Anna Maria Zicari, Marzia Duse, Alberto Spalice

**Affiliations:** ^1^Child Neurology Division, Department of Paediatrics, “Sapienza” University of Rome, Rome, Italy; ^2^Child Immunology Division, Department of Paediatrics, “Sapienza” University of Rome, Rome, Italy; ^3^Child Cardiology Division, Department of Paediatrics, “Sapienza” University of Rome, Rome, Italy; ^4^Department of Internal Medicine, “Sapienza” University of Rome, Rome, Italy

**Keywords:** PANDAS (Pediatric Autoimmune Neuropsychiatric Disorders Associated with Streptococcal Infection), heart murmurs, cardiologic consultation, tics, *Streptococcus* beta hemolytic, PANS (Pediatric Acute-onset Neuropsychiatric Syndrome), mitral valve (MV), pediatry

## Abstract

**Objective:** Cardiac involvement in PANS has not been clarified relying on the scientific literature available until today. It is known that streptococcal infections play a role in the etiology of a great number of diseases including Sydenham chorea and rheumatic fever, among others. Based on the suspected pathogenesis of PANDAS (Pediatric Autoimmune Neuropsychiatric Disorders Associated with Streptococcal infections) reported in the medical literature, we decided to investigate the cardiologic involvement in children with a recent PANS/PANDAS diagnosis.

**Methods:** The study population satisfies PANS (1) and PANDAS (2) criteria of diagnoses. Cardiologic assessment was performed through clinical examination, electrocardiography, and echocardiography.

**Results:** In the selected pediatric population, a significant number of children presented mitral valve involvement, systolic murmurs and electrocardiographic abnormalities. High ASLOT levels did not seem to be associated to a cardiac involvement.

**Conclusions:** Often PANS is difficult to diagnose because it is little known by physicians and most of the cardiologic findings described in this study are common among the healthy pediatric population. Also, ASLOT levels seems not to be predictive of cardiac involvement. Furthermore, the existence of PANDAS as a clinical entity is associated with a group of anti-neuronal autoantibodies found in Sydenham chorea is still controversial. We recommend a complete cardiologic evaluation in those children who meet the PANS/PANDAS diagnostic criteria.

## Aims

Over the past 20 years, pediatric autoimmune neuropsychiatric disorders associated with streptococcal infections (PANDAS) and a group of anti-neuronal autoantibodies, which signal neuronal cells in the basal ganglia, have emerged as a new disease (3, 4). Although the diagnostic criteria are clear, it is still a difficult diagnosis which is based only on the clinical examination of symptoms, so it remains a controversial diagnosis. For this reason, the description of PANDAS has been modified to eliminate etiological factors and to designate an expanded clinical entity: pediatric acute-onset neuropsychiatric syndrome (PANS) or idiopathic childhood acute neuropsychiatric symptoms which deserves further study (5). Furthermore, the cardiologic involvement has never been studied in detail. Here, we report our experience with children recently diagnosed with PANS/PANDAS. We investigated the presence of cardiologic signs through clinical examination, electrocardiography (ECG) and echocardiography. We compared these results with the general pediatric population according to the literature and we also studied the possible association with anti-streptolysine O titer (ASLOT) levels.

## Introduction

**Figure 1:**
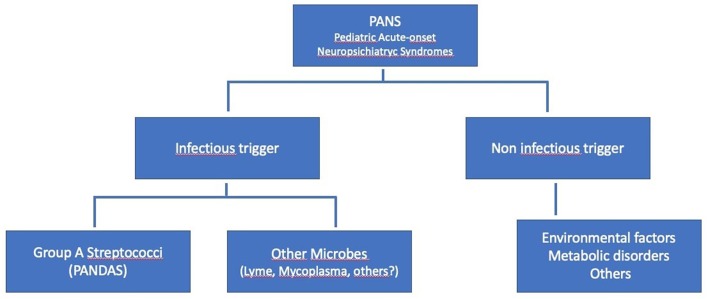
Hierarchy of the Pediatric PANS, modified from Swedo et al. (1).

Pediatric acute-onset neuropsychiatric syndrome (PANS) or idiopathic childhood acute neuropsychiatric symptoms are emerging in the last few years as a new clinical entity. Some of these clinical pictures could be grouped into what represents a sub-group of PANS, better known as PANDAS, which represents, at least in part, an attempt to provide a hypothesis about the origin of this symptoms complex. In other words, the concept of PANS is relatively recent and it is derived from subsequent researches on PANDAS, which now is considered like a specific subset within the broader clinical spectrum of PANS (6, 7) (see Figure 1).

The potential mechanisms for these diseases are known (8) and the first studies about the correlation between streptococcal infections and many clinical features [like streptococcal M protein and rheumatic fever (9) or Streptococcal antibody titers in Sydenham's chorea (10)] took place in the sixties and seventies (11, 12). In the last years some studies seem to identify a pathogenic trigger of autoimmunity in acute rheumatic fever and streptococci (9, 13). In particular Group A streptococcus (GAS) carbohydrate is considered as an immunogen (14, 15) and the crossreactive antibodies were proven by Cunningham et al. (4, 16, 17) using human and mouse monoclonal antibodies (18–21). The cause of streptococcal sequelae is well-known, and PANS/PANDAS is most definitely a streptococcal sequelae and has a group of anti-neuronal autoantibodies that are identical to those found in Sydenham chorea (22, 23). Crossreactive antibodies produced against the group A streptococcus and heart and brain and molecular mimicry are the causes of these, with many studies to support this hypothesis (4, 16, 17, 19, 21–23).

Pediatric autoimmune neuropsychiatric disorders associated with streptococcal infections (PANDAS) describes a set of signs and symptoms including obsessive compulsive disorders (OCD), verbal or motor tics or cognitive disorders in children with a history of group A beta-hemolytic streptococcus Group A streptococcal (GAS) infections, which are known to lead to the induction of a group of anti-neuronal autoantibodies (24, 25), as well as autoantibodies against the heart valve (26). So, as just seen, the idea that there are crossreactive antibodies and that molecular mimicry is the underlying basis of initiation of rheumatic fever is strongly supported.

PANDAS is diagnosed in children from 3 to 14 years old. It affects both males and females but is more common in males (2.6:1). According to some studies of children in this age group (27, 28), PANDAS children produce antibodies against the M protein, a component of the bacterial GAS wall (29). So, the disorder involves the classic exacerbations of GAS infections (30) and afterwards manifestation of tics and OCD (31).

It is difficult to establish a consensus on the epidemiology of this syndrome. Today, after 30 years of research, PANDAS is still not a well-known disease, and it is likely that many children are undiagnosed. The incidence and prevalence of this pathology are not available because PANS/PANDAS is a new pathology which is still being studied today and is probably underestimated by physicians. There is no recent data on the general prevalence of tics and OCD worldwide in 2018; however, there is a trend toward increased diagnosis in the pediatric population thanks to better awareness among pediatricians. According to Knight et al., the prevalence of tics and OCD in the pediatric population of the USA was 2–3% in 2012 (32). Transient tics are common among school-age children, with estimates ranging between 4 and 24%, with the number of children with chronic tics roughly one-quarter of this number. The prevalence of tic disorders peaks late in the first decade of life through to early in the second decade due to the clinical course of the disorder. The prevalence of tic disorders is roughly one-third of this number in adulthood. Boys are twice as likely as girls to be affected by tic disorders (5). Results from the Epidemiological Catchment Area study, which assessed more than 18,500 adults with structured diagnostic interviews, estimated the lifetime prevalence of OCD among adults to be between 1.9 and 3.3%. Epidemiological studies of adolescents provide similar estimates in the range of 1.9–3.6%. The sex distribution of these epidemiologic studies suggests that OCD affects males and females equally after puberty. However, males have an earlier age of onset than females, with their initial presentation typically occurring well before puberty (33–35). PANDAS is difficult to diagnose because its features (i.e., tics, OCD, history of GAS) have a high prevalence among the pediatric population.

The patient's clinical history related to clinical symptoms is very important for the diagnosis, even better if supported with videos showing tics and OCD, which are not always present during clinical evaluation. In many cases, a correlation has been found between GAS post-infection tics and cardiac abnormalities, with mild mitral valve involvement (1, 2, 27, 28, 36, 37).

It is known that GAS plays a role in the etiology of a great number of diseases and sequelae, including Sydenham chorea and rheumatic fever (16, 38), among others.

The heart valve involvement can be observed with Sydenham chorea and this is often the case to see a murmur or rheumatic carditis with Sydenham chorea (24–26); since the moment that it represents a characteristic pattern the authors would be looking at PANS/PANDAS for valve disease because its similarity to Sydenham chorea which presents also the same anti-neuronal autoantibodies. Clearly murmurs appear in patients with Sydenham chorea so it is not unreasonable to believe they would be associated with PANS and PANDAS and elevated ASLO titers.

With regard to Acute Rheumatic Fever (ARF), the ASLO is positive in many cases as it is present and part of the Jones criteria, but it should be considered only when one major and two minor Jones criteria are present, according to the most recent Revision of the Jones Criteria for the diagnosis of acute rheumatic fever (39). Clearly it is known that the ASLO can be elevated many folds, with a great variability, and ARF has no relationship to strength of titer. There are cases of valve disorder that have ASLO positive titers. It is yet known that 40% of cases of pharyngitis or any streptococcal disease or sequelae are not positive in the ASLO. The ASLO needs to be supplemented with throat cultures or the anti-DNASe B or the streptozyme assay for 5 anti-streptococcal antibodies. These extra assays are often not done so usually 40% of the cases may be missed. The quick strep test is often used in cases to determine pharyngitis.

In the “Revision of the Jones Criteria for the diagnosis of acute rheumatic fever in the era of Doppler echocardiography: a scientific statement from the American Heart Association” (39), Gewitz et al. stated the echocardiographic criteria of rheumatic carditis and in particular the echocardiographic criteria for the diagnosis of pathologic regurgitation of mitral and aortic valves. Thus, although moderate or severe mitral insufficiency could be included in this classification, mild mitral regurgitation would be excluded.

Due to the fact that the real cause of PANS is unknown and based on the suspicious pathogenesis of PANDAS reported in the pediatric literature, we decided to investigate the cardiologic involvement in children with a recent PANS/PANDAS diagnosis. Due to the ongoing controversy regarding PANDAS, there has been an increased effort to establish a more rigorous definition of the clinical phenotype for pediatric acute-onset neuropsychiatric syndrome (PANS), while leaving open the potential etiological role of antecedent GAS infections or other post-infectious inflammatory processes. The cardinal feature of PANS is the sudden onset (within 24–48 h) of OCD or a restrictive eating disorder, accompanied by two or more sudden-onset neuropsychiatric symptoms (1) and anti-neuronal autoantibodies which target the basal ganglia (3).

The hypothesis of this study arises from some clinical observations reported below. It is well-known that ECG changes occur during and after streptococcal infections in about 50% of the cases of pharyngitis but these changes usually are transient. So a similar phenomenon can be hypothesized regard to a transient valve disease or lower grade murmurs that may eventually become problems in later life for the adult. The basic mechanism would be expected to be the well-known antibodies against the group A carbohydrate that are known to affect the valve in prognosis of rheumatic valvular heart disease (10).

Thus, the explanation of why the authors are looking at this topic, and in particular at this children, in the first place is because of rheumatic heart disease after streptococcal infections which is the case in PANS/PANDAS in our cohort with 76.7% ASLOT positive.

## Study Design

The study population consisted of 30 children of between 6 and 15 years of age (mean age 9.23 years; 26 males and 4 females, male-female ratio 6.5:1) enrolled during 2018, who had been previously diagnosed with PANS/PANDAS (mean age at diagnosis 8.53 years).

**Figure 2:**
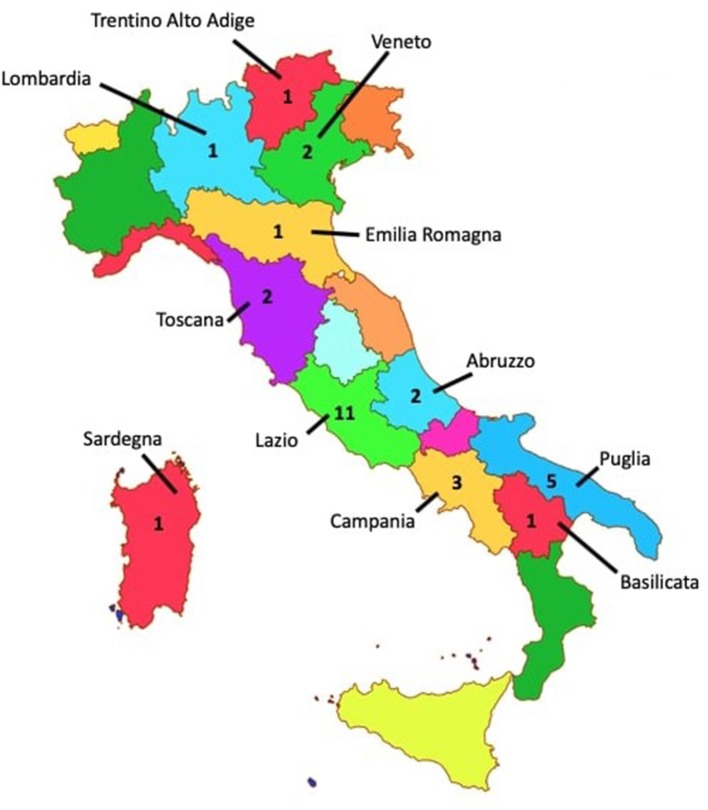
Map of the 20 Italian regions with number of patients per region (zero where not indicated). In accordance with the diagnostic criteria for PANDAS established by Swedo et al. (2), we selected children with the following characteristics:
**- Presence of OCD and tics** according to the diagnostic criteria for tics and OCD of the DSM-IV and DSM-V;**- Onset in childhood** (from 3 years to puberty);**- Sudden onset and cyclic exacerbation of more severe symptoms**, as the patient's parents consult doctors when the child begins to show tics, OCD and other pathological behaviors, or a return of past neuropsychiatric symptoms;**- Temporal connection between symptom onset and GAS infection** [positive pharyngeal swab, high anti-streptolysin O titer; ASLOT (2)], as sometimes the infection has progressed by the time of disease onset (also 6–9 months), although most children experience symptom onset at 6 weeks;**- A connection with neurological abnormalities**, including cognitive difficulties, worsening academic performance, dysgraphia, ADHD, depression, separation anxiety, irritability, emotional lability, oppositive behavior, sleeping difficulties, choreiform movements, and Romberg test abnormalities.

The ethnicity of all of the patients was Caucasian, and all of them are born in various regions of Italy (see Figure 2). Data about socio-economic status of the patients' families were not collected. Patients who were <18 years old have been enrolled if they satisfied PANS (1) and PANDAS (2) criteria of diagnoses.

The patients have been also enrolled in this study according to Swedo et al. that modified the PANDAS criteria to describe PANS in 2012 (1) as follow:

- **Abrupt, dramatic onset of obsessive-compulsive disorder or severely restricted food intake**.
- **Concurrent presence of additional neuropsychiatric symptoms**, with similarly severe and acute onset, from at least two of the following seven categories:

1. Anxiety;
2. Emotional lability and/or depression;
3. Irritability, aggression, and/or severely oppositional behaviors;
4. Behavioral (developmental) regression;
5. Deterioration in school performance;
6. Sensory or motor abnormalities;
7. Somatic signs and symptoms (including sleep disturbances, enuresis or urinary frequency).

- **Symptoms are not better explained by a known neurologic or medical disorder, such as Sydenham chorea, systemic lupus erythematosus, Tourette disorder or others** (1).

Other major conditions represent exclusion criteria and have been excluded through targeted diagnostic analysis. Some of the most important conditions excluded are acute rheumatic fever [according to the revised Jones criteria (39)], rheumatologic diseases, immunologic impairment, anti-phospholipids syndrome, acute or chronic infections, acute pharyngitis, encephalitis, meningitis, presence of auto-antibodies in a blood sample, etc.

All patients had a negative culture of the pharyngeal swab at the time of the enrollment.

A part tics and OCD, which represent the main reason why parents consult a pediatric neurologist in this circumstance, and other psychiatric conditions were excluded during the medical interview, medical history and medical examination.

Only one of the patients reported a previous diagnosis of Wolf-Parkinson-White syndrome. None of the other selected patients report relevant disease in their medical history.

A standard screening panel was applied to each patient enrolled in the study and included: complete blood count with formula, basic screening coagulation, biochemical blood tests, extended auto-antibodies panel, immunoglobulin count, acute phase reactants, pharyngeal swab.

The parents of all patients read and signed a written informed consent to the study before they could be enrolled.

According to our study protocol, the patients were submitted to general examination, neurological examination and cardiological examination with electrocardiography (ECG) and echocardiography. All tests and examinations were performed by expert specialists.

A General Electric Healthcare Vivid^TM^ E9 with XDclear^TM^ cardiovascular ultrasound system was used to perform all the echocardiographs.

Some of the patients were yet to be treated with antibiotics (azithromycin, cephalosporin, or penicillin) or antipsychotics (haloperidol or risperidone). At the time of data collection, 27 out of 30 patients (90%) were undergoing antibiotic therapy, and six patients (20%) were undergoing other therapies (haloperidol or risperidone), which they had started within the past 6 months. All of the patients presented tics or OCD, and these symptoms were the reason that their parents had originally brought them to our center for medical observation. Among our population, only two children had undergone previous cardiologic examination in another center, with one diagnosed with Wolf-Parkinson-White syndrome (WPW).

## Results

**Figure 3:**
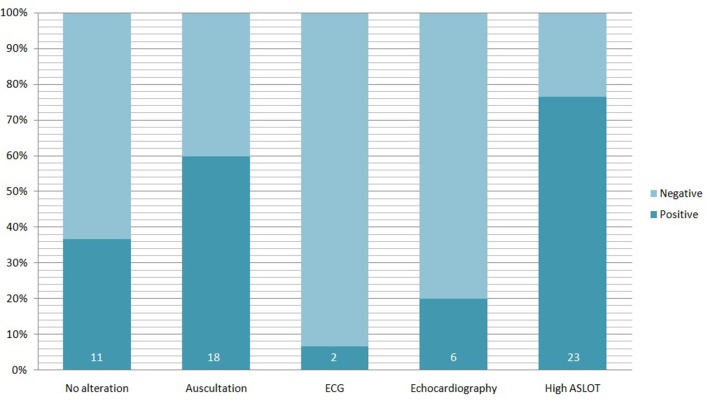
Alterations founded in 30 pediatric PANS/PANDAS patients. Percentage refers to the totality of the study population; the darker bars represent the percentage of positive patients for each test (the number inside indicate the absolute number of positive patients).

During the cardiologic examination, 17 out of 30 patients (prevalence 56.7%) were positive for cardiac systolic murmur under auscultation (1/6° Levine scale), and one (3.3%) showed inconsistent doubling of II cardiac tone (see Figure 3).

All patients underwent cardiac ultrasonography by an expert cardiologist, who found oval foramen patency (POF) in a patient (3.3%) and mild mitral valve insufficiency in 5 patients (16.7%). In the ECG a single patient showed sharp T waves in V1 and V2 derivations, instead the child with yet known WPW syndrome showed ventricular pre-excitation.

In the pediatric population at our center diagnosed with PANS/PANDAS, according to the criteria published by Swedo et al., we found a significant prevalence of cardiac involvement, analyzed both clinically and instrumentally using ECG and cardiac trans-thoracic ultrasound (see Table 1).

**Table 1:** Patients characteristics and data collected.

**Patients**	**Data**
Total	30 patients
Males	26 patients (86.7%)
Females	4 patients (13.3%)
Males: Females Ratio	6.5:1
Age	Mean 9.23 years; range 6–15 years
Age at diagnosis	Mean 8.53 years; range 5–15 years
Antibiotic therapy	27 patients (90%)
Time from starting antibiotic therapy	Mean 10 months; range 6–12 months
Other drugs	6 patients (20%)
Normal cardiologic findings	11 patients (36.7%)
Cardiac auscultation abnormalities	18 patients (60%)
ECG abnormalities	2 patients (6.6%)^*^
Echocardiography abnormalities	6 patients (20%)^**^
High anti-streptolysine O titer (ASLOT)	23 patients (76.7%)

* *One patient with a previous diagnosis of Wolf-Parkinson-White Syndrome*.

** *One patient with oval foramen patency (POF)*.

ASLOT was considered normal for values between 0 and 166 IU/l, altered for higher values. Mean value among the study population was 395 IU/l, 330 IU/l among the patients with cardiologic involvement and 492 IU/l in those who present a normal cardiologic screening. In our study cohort, 23 on 30 children (76.7%) presented with an elevated ASLOT. In this sub-group 11 on 23 (47.8%) presented also a cardiac involvement. We can also consider that 11 patients over 17 (64.7%) with cardiac involvement presented with an elevated ASLOT. Twelve of 13 patients presented without cardiologic involvement and a high ASLOT (92.3%). Table 2 resumes the correlation between cardiologic involvement and ASLOT values in the studied population.

**Table 2:** Distribution of cardiac involvement and ASLOT in the study PANS/PANDAS patients.

**Patients**	**Normal ASLOT**	**High ASLOT**	**Total**
Cardiac involvement	6	11	17
No cardiac involvement	1	12	13
Total	7	23	30

*Referring to these data we can calculate the relative odds ratios as state in the sections “Study design” and “Conclusions”*.

The Odds Ratios (OR) have been calculated in order to determine the strength of association between the presence of cardiologic involvement and high ASLOT. The risk of finding a cardiologic involvement in the group with high ASLOT instead of a normal ASLOT correspond to an OR of 0.153. The risk of finding an elevated ASLOT in patients with cardiologic involvement respect to the patients with normal cardiologic screening corresponded to an OR of 0.153.

In conclusion, ASLO strength of titer is not associated with the cardiologic findings in patients or normal populations.

## Discussion

Quoting the DSM-5 (40): “Although there is a body of evidence that supports the existence of PANDAS, it remains a controversial diagnosis. Given this ongoing controversy, the description of PANDAS has been modified to eliminate etiological factors and to designate an expanded clinical entity: pediatric acute-onset neuropsychiatric syndrome (PANS) or idiopathic childhood acute neuropsychiatric symptoms which deserves further study”. These diseases, as well as Sydenham chorea, have been associated with a group of anti-neuronal autoantibodies against the basal ganglia (24, 25).

Until now, a clear correlation between PANS and cardiological alterations has not been demonstrated (36, 37, 41). On the other hand it is well-known that streptococcal infections play a role in the onset of a certain number of streptococcal sequelae such as Sydenham chorea, rheumatic fever, which can affect the nervous and/or cardiovascular system through mechanisms of molecular mimicry where anti-streptococcal antibodies against the group A carbohydrate are directed against heart valve and/or basal ganglia (23, 24, 26). Keeping these facts in mind, based on similarities, the authors of this study propose PANDAS as a possible (but not proven) hypothesis in order to explain the significative correlation between PANS and cardiological findings in the population of the study.

In the selected study population, a significant number of children presented with mild mitral valve involvement, systolic murmurs and ECG abnormalities. Based on this evidence, we recommend a complete cardiologic evaluation in those children who meet the PANS or PANDAS diagnostic criteria.

Most of the cardiologic findings described in this study are common along the healthy pediatric population, and they can act as confounding factors. PFO and WPW are incidental findings from congenital origin, unrelated to inflammatory diseases. Further, high ASLOT positivity, common in children, should only be considered in those patients who present with tics, OCD, and valve alterations. Murmurs are the most common reason for pediatric cardiologist referral and evaluation in the general pediatric population. There are reports that up to 72% of children will have a murmur at some point during childhood and adolescence. Of these, some may persist until adulthood, while others will resolve over time. The incidence of congenital heart disease in the general population is <1%, and of all new murmurs referred to a pediatric cardiologist, <1% are the result of congenital heart disease (33).

The authors of this study are aware of only a few data in the literature about cardiac involvement in PANS patients (36, 37) which has never been clarified. As the PANS and PANDAS diagnoses are currently underestimated, not well-understood and not commonly known, the size of the statistical sample is limited. Furthermore, in our study the patients who presented a valvular insufficiency had a mitral valve regurgitation defined as “mild,” so they did not meet either the revised Jones criteria nor the echocardiographic criteria of rheumatic carditis or pathologic mitral regurgitation (39).

Among the study population 76.7% of patients presented with high ASLOT. In the study of associations, it seems that patients who presented with high ASLOT are not associated with an abnormal cardiologic screening compared to the group with normal ASLOT levels (OR = 0.153). In the same way the presence of cardiac involvement among patients is not associated to higher levels of ASLOT compared to the group of patients without cardiologic abnormalities (OR = 0.153).

ASLO titers were studied in comparison to the valve alterations. The Odds Ratio was 0.153 and it was previously stated that it indicates no association with the ASLO titer. This is the same as in acute rheumatic fever where there is no association with the elevation of the titer. The valve alterations or low grade murmur that persists or disappears over time is likely due to streptococcal infection. The study bringing attention to the subclinical and less severe types of valve disease in both “normal” and PANS/PANDAS children.

Although there is only one animal model of rheumatic heart valve disease at the current time, which is the Lewis rat model originally published in Infection and Immunity by Quinn (8) and Cunningham et al. (16, 19–21), the authors hypothesize that the same mechanism may be the potential cause of these minor valve problems or events in normal children, which may go away or permanently persist. Our study suggests that transient or permanent low level damage may take place in children after streptococcal infections, and we assume that it may happen through a mechanism based on autoimmunity and molecular mimicry with the with the formation of autoantibodies that may lead to these valvular alterations associated with streptococcal infections. The autoantibodies known to affect the valve may play an important role in this instance and cause this subclinical and low grade murmur that would be considered lower end of the spectrum of valve disease in children and might disappear or persist.

Also, the ECG changes that occur during and after streptococcal infections in about 50% of the cases of pharyngitis, which are usually transient, make us think that transient valve disease or lower grade murmurs may eventually become problems in later life for the adult. The mechanism would be expected to be antibodies against the group A carbohydrate that are known to affect the valve in prognosis of rheumatic valvular heart disease, as already established earlier in this article.

The fact that streptococcal infections have long been associated with valvular heart disease suggests that subclinical and lowgrade forms of disease could also be present, and it is highly likely that they do occur, but this has never gained any attention. Not all of the children had greatly elevated titers but because the ASLO cannot reliably detect all cases of streptococcal infections, throat cultures and other tests can be used to increase the evidence that these were associated with streptococcal infections. The ASLO however was elevated in 76.7% of the cases and if other tests had been used for streptococcal infections at the time of disease then this would likely increase. The presence of lower level valve disease in PANS/PANDAS is very interesting and also brings the disease closer to a milder form of rheumatic fever and this could change the point of view and the approach concerning this disease. For years there has been no hypothesis about childhood mild or subclinical valvular disease, transient or persistent, and now there can be this hypothesis.

Due to the lack of studies on cardiologic aspects of the pediatric population with PANS/PANDAS, we suggest that a cardiologic screening should be performed at the onset and a cardiologic follow-up at least once every 1 year until puberty. However, further studies are needed to improve our knowledge on this subject. Longitudinal studies could be useful to allow determination of any progression of heart involvement after the initial diagnosis.

## Table of Contents Summary

The cardiologic involvement in PANS has never been studied in detail. We investigated the presence of cardiologic signs through clinical examination, electrocardiography and echocardiography.

## What's Known

PANS describes signs and symptoms, like obsessive compulsive disorders, verbal or motor tics or cognitive disorders. PANDAS is a subset of patients who develop PANS symptoms triggered by an infection; specifically a streptococcal one. Streptococcal infections are involved in the pathogenesis of many diseases, including Sydenham chorea and rheumatic fever.

## What's Added

Based on the definition of PANS and the suspected pathogenesis of PANDAS reported in the medical literature, we decided to investigate the cardiologic involvement in children with a recent PANS/PANDAS diagnosis and their association with ASLOT levels.

## Data Availability Statement

The datasets for this manuscript are not publicly available because these are the result of the analysis of the analyzed data collected in the DH Pandas. Requests to access the datasets should be directed to alberto.spalice@uniroma1.it.

## Ethics Statement

This study was carried out in accordance with the recommendations with written informed consent from all subjects parents. All subjects parents gave written informed consent in accordance with the Declaration of Helsinki. The protocol was approved.

## Author Contributions

DB, MM, and AS coordinated and supervised data collection, conceptualized and designed the study, reviewed and revised the manuscript. All authors: (a) gave substantial contribution to conception and design, acquisition of data, or analysis and interpretation of data; (b) drafting the article or revising it critically for important intellectual content; (c) gave the final approval of the version to be published; (d) agree to be accountable for all aspects of the work in ensuring that questions related to the accuracy or integrity of any part of the work are appropriately investigated and resolved.

### Conflict of Interest

The authors declare that the research was conducted in the absence of any commercial or financial relationships that could be construed as a potential conflict of interest.
